# Generative artificial intelligence in jazz improvisation: an Actor-Network Theory perspective

**DOI:** 10.3389/fpsyg.2026.1895145

**Published:** 2026-10-01

**Authors:** Chenge Gao

**Affiliations:** Popular Music Studies, Department of Musicology, Shanghai Conservatory of Music, Shanghai, China

**Keywords:** Actor-Network Theory, agency, empirical musicology, generative artificial intelligence, jazz improvisation

## Abstract

Generative artificial intelligence (GAI) plays an increasingly significant role in the artistic creation process. In the domain of highly interactive human-machine composition, GAI is no longer a passive tool but exhibits a certain degree of agency. Based on Actor-Network Theory (ANT), a social science theoretical framework proposed by French sociologist Bruno Latour and other scholars, GAI can be viewed as a non-human subject endowed with the status of an actor. In jazz improvisation, a representative creative activity, the agency of GAI is manifested in the generation of its specific musical works, presenting a contrast to human creation. Furthermore, this process reveals the leading and constraining factors in the actions of GAI, as well as the potential pathways and mutual interactions of its future co-creative development with humans.

## 1 Introduction

We are in the midst of an unprecedented era of change, where generative artificial intelligence (GAI) is reshaping the world at a speed beyond imagination. The transition of technology not only alters lifestyles but also serves as the starting point for a cognitive paradigm shift. In our social and musical lives, just as the invention of the phonograph eliminated the absolute dependence of music on the human physical presence and profoundly transformed musical ontology (Hogg, 2012), this poses a radical challenge to any interdisciplinary détente, necessitating the establishment of a meta-analytical framework that understands recorded heritage as constitutive of all musical experience over the last century (Born, 2010). The intervention of GAI undoubtedly demands new methodologies for observation, finding a methodology that fits the current media environment is particularly critical. As concepts such as technology, creativity, and authenticity become highly intertwined in the musical domain, the musical practice of GAI inevitably becomes the center of controversy.

Whether GAI possesses a creative soul, can express emotions exclusive to organisms, and whether it can bridge the embodiment gap remain profound questions. If a negative assertion is directly given, GAI can easily be simplified into a passive, mechanical, dichotomous tool, rendering it incapable of addressing various current issues. Therefore, adopting an empirical program seems to be an appropriate path: it neither claims that humans and artifacts are completely identical, nor does it argue that they are entirely distinct (Callon and Latour, 1992). Actor-Network Theory (ANT) is a methodology rather than a topology, it does not go looking for network-shaped things but rather attempts to register the effects of anything that acts in a given situation, regardless of whether that actor is human, technological, discursive, or material (Piekut, 2014). This is a more ambitious and unified attempt at object-oriented cultural analysis, which has taken shape in cultural studies fields adjacent to the social sciences. Characterized by theoretical models dedicated to eliminating or weakening the fundamental differences between objects and humans, the core argument of such research is that objects and humans possess roughly equal degrees of agency and methodological importance (Straw, 2012). Action is no longer perceived as the sole realm of the human actor, but also the realm of the non-human actor, including the technological artifact. By virtue of their presence, the world is not exactly as it was before, a positivity has been made that changes the course of events (Prior, 2008). It is precisely this actor-network—which transcends anthropocentrism and treats technological artifacts as a novel type of agentic subject—that provides us with a heterogeneous theoretical horizon to re-examine musical practices. Within this framework, improvisation, as the most intense and dynamic arena for human-machine interaction and semiotic translation, spans a scope of association and a breadth of referential meaning that allow the underlying cultural narrative to unfold.

Improvisation studies have witnessed an explosive growth in recent years, and this interdisciplinary critical perspective has long transitioned beyond pure musicological boundaries, extensively penetrating into the sciences, arts, and humanities. Many non-music scholars are utilizing improvisation as a core model to profoundly intervene in the interdisciplinary construction of diverse, grand issues such as social identity, computational creativity, embodied cognition, distributed agency, and even the histories of peoples and nations (Lewis, 2013). This demonstrates that improvisation is not merely a musical phenomenon, but a fundamental aspect of human activity. In past musical practices of human-machine interaction related to improvisation, there are many representative cases and relevant discussions. For instance, George E. Lewis demonstrated in his computer interactive system *Voyager* that AI is no longer viewed as an objective or universal instruction executor but is reconstructed as a conversational subject embedded with specific ideas, thought communities, and cultural features of its creators (Lewis, 2000). With further technological evolution, the SoVo system integrates the phrase generator Voyager with Somax2, which is trained on a musical data corpus, becoming an architecture that can both interact with external participants and operate autonomously. This also propels human-machine collaboration into a posthuman dimension, in this practice, becoming a player is not a human attribute or intent but a process of entering into an assemblage where the boundaries between the performer, AI, and shared sonic environments dissolve into an emergent, collective sonic assemblage, enacting a new mode of ubiquitous, posthuman creativity where ego and subjectivity disappear and the assemblage plays itself (Fiorini et al., 2026). Improvisational creative performance can be regarded as the creative process in microcosm. Observing improvisational creative performance provides valuable predictions and important references for creative fields in which the creative process takes too long to directly observe. The study of jazz performance as a “visible creative process” is particularly helpful in understanding aspects of the individual creative process that are salient in improvisational creativity but elusive in compositional creativity (Sawyer, 1992).

Based on the theoretical context outlined above, this paper aims to conduct a comparative study within the ideological horizon of ANT, examining three distinct modes of jazz improvisation: the human performance recordings of Thelonious Monk, Shimon's live robotic improvisation, and Suno's digital generation. By synthesizing empirical and musicological analyses, this research elucidates the expressive characteristics of GAI in jazz improvisation. On this basis, it critically scrutinizes the evolving human-machine relationship in collaborative artistic production, attempting to transcend mere simulation and move toward a posthuman mode of co-creation. This paper contends that while ANT remains the most direct and potent tool for dissecting the dynamic interactive networks of GAI, it by no means holds an interpretive monopoly. Given the theoretical deficiencies of ANT when confronting ideologies and structural exclusions, this study strategically integrates Georgina Born's perspective of relational musicology, reflecting on how GAI participates in translation as a materialized ideological mediation, as well as the unequal social realities mapped out beneath its surface.

## 2 Overview of Actor-Network Theory

Actor-Network Theory (ANT) is a social science theoretical framework that emerged in the mid-1980s, centering on the theoretical contributions of the renowned French philosopher, sociologist, and anthropologist Bruno Latour. It redefines the “social” as a dynamic process of connectivity rather than a fixed domain or a specific substance. Latour entered the field of science studies in the late 1970s, utilizing anthropological fieldwork methods to study the sociology of scientific knowledge. Latour proposed that the construction of social relations cannot be explained merely through the following simplified approaches: namely, the material infrastructure that determines social relations, as in Marxist materialism; a mirror reflecting social differences, as in Pierre Bourdieu's critical sociology; or a scenic background for human social actors to play the leading roles, as in Erving Goffman's interactionist theory (Latour, 2005). Latour argued that while these perspectives are not entirely incorrect, they are oversimplified and fail to comprehensively describe the complex relationships between humans and non-humans. In reality, society and the collective are composed of multiple intertwined associations, requiring more complex and nuanced theories to understand. What science studies discusses is not the relationship between social context and the interests of different social forces, but rather their interconnections with collectives and objects (Latour, 1993).

Based on this, after the 1980s, Latour, along with scholars such as Michel Callon and John Law, co-developed ANT, resolving the contradiction between material and social construction through the extension of anthropology and semiotics. The core of ANT is to demonstrate that societies, organizations, agents, and machines are all effects generated within patterned networks composed of diverse, non-purely human materials (Law, 1992). ANT provides a decentralized perspective for the social sciences, challenging the traditional binary oppositions of subject-object and society-nature. By emphasizing connectivity and agency, it deconstructs complex social phenomena into dynamic combinations of relations, offering new methods for investigating social and technological interactions and contributing significantly to the understanding of how science and technology shape social reality.

Within the paradigms of traditional metaphysics and ethics, action and agency were inherently positioned within a substantial framework characterized by mental-physical duality and volitional centrality. Traditional philosophy presupposed that an agent must be a particular object or substance of a specific kind, historically and exclusively prioritizing the human person as its paradigm—a substance that simultaneously possesses both physical properties and mental properties. Along this classical lineage, John Locke conceptualized the “will” as an “active power” of the mind, contending that action is essentially driven by internal volitions (Locke, 1690/1975). Subsequently, Thomas Reid established the foundational pioneering status of agent-causation, emphasizing that the agent *per se* operates as an independent, primary efficient cause capable of breaking physical stasis and actively exerting a first pushing force (Reid, 1969).

As the twentieth century advanced, the philosophy of action bifurcated from this macro-philosophical matrix, shifting toward a microscopic deconstruction of the ontological status and logical form of action itself. Within this modern domain, the agent and the patient are commonly described as possessing, respectively, an active power and a corresponding passive power (or liability); thus, the agent's action and the patient's reaction constitute the manifestation or exercise of their respective powers on particular causal occasions. Ontologically speaking, action should not be viewed as a pure event. Token actions, as E. J. Lowe determinately argues, are quite simply a sub-class of particular properties—namely, tropes or modes—which are fundamentally distinguished by the fact that they are concrete manifestations or exercises of the very “powers” possessed by the agent on specific occasions (Lowe, 2010).

In the actor-network, Latour positions “actor, translation, network” at the core. Departing from the emphasis on the subject in previous theories of action, actors include not only humans but also technologies, objects, animals, and other non-human entities. Actors and networks are mutually constitutive within the actor-network; the identity and role of each actor are determined through its relations with other actors, and these relations themselves form the network. The network is not a background, but the core of social phenomena. The actor-network breaks down the boundaries between humans and non-humans in traditional social sciences, proposing that non-humans also possess agency, which is defined as the capacity of an actor to alter states or make a difference (Latour, 2005). In other words, objects, technologies, and other non-human factors play an equally vital role in social interactions; they are not merely tools or media but exert real effects on social processes. Non-humans are presented in a new light within social theory, they must be treated as actors rather than mere vehicles of symbolic meaning. Action is multi-layered and prone to concealment, constituting a node made of many complex agencies that need to be progressively deconstructed.

## 3 The conceptual origins and technical iterations of GAI

As early as the 19th century, Ada Lovelace, a pioneer of computer programming, prophetically anticipated the profound connection between computers and music while writing forward-looking notes on the Analytical Engine. In her famous “Note G,” she originally pointed out that if the harmonic nature and expressive laws of pitches could be scientifically abstracted, the Analytical Engine could utilize them to compose and arrange scientific music of arbitrary complexity and scale. However, constrained by the historical conditions of her era, Lovelace maintained a mechanistic prudence when facing the creative boundaries of technological artifacts, arguing that the Analytical Engine has no pretensions whatever to originate anything and can only do whatever we know how to order it to perform (Menabrea and Lovelace, 1843). This classic dictum regarding the machine's inability to innovate spontaneously and its subordination to command was deconstructed across generations a century later by Alan Turing. In discussing computing machinery and intelligence, Turing dedicated a specific section to offer a powerful response to this “Lady Lovelace's Objection.” Drawing from his own practical experience operating early computers, Turing pointed out that machines take us by surprise with great frequency, and sharply deconstructed the anthropocentric myth of originality: Who can be certain that the original work he has done was not simply the growth of the seed planted in him by teaching, or the effect of following well-known general principles (Turing, 1950)?

This ideological confrontation spanning two centuries essentially outlines the ultimate goal of the development of computational creativity, positing that robots must possess the capacity to generate spontaneous ideas to be deemed intelligent. In the realm of music, machines can apply randomly generated numbers, formulas, and logics to arrange arbitrary notes, thereby producing countless original melodies, yet those that are truly pleasing to the ear are few and far between. Conversely, shaping a natural creative process that evolves through algorithms might grant machines the possibility of creating precious artworks possessing authenticity, from which the goal of AI arises. GAI, fundamentally anchored in large language models, has broken through the technological paths of traditional AI, demonstrating disruptive generalization capabilities and autonomous interactive abilities across various fields of human-machine interaction, forging a brand-new human-machine relationship, and simultaneously triggering fierce debates on critical issues related to the development of human civilization.

Within the actor-network, GAI is by no means merely a tool designed by humans or a passive technology, but rather an actor endowed with agency. As an important subclass of AI, GAI not only executes commands but also alters states within social and technological networks through the generation of content. In this process, GAI influences the ecology of creative industries, such as subverting the workflow of artistic creation and endowing “originality” and “creativity” with new definitions. The actor-network framework emphasizes the mutual shaping of actors and networks, meaning that the network constitutes the identity and agency of the actors, while the actions of the actors conversely reorganize the network. This co-constitutive relationship is particularly prominent in the interaction between humans and GAI, where factors such as hardware capabilities, data quality, and algorithmic optimization influence the performance of GAI, which in turn shapes the behavior and cultural cognition of human users. In jazz improvisation, a musical form rich in creative practice, the “composition” of GAI is concretely manifested. As a participant, GAI does not merely “generate” music, but acts as an actor in the process of musical creation, intervening in the construction of jazz improvisation and forming coordination and collaboration with human musicians.

## 4 Jazz improvisation studies and analytical methods

Improvisation, referring to unpremeditated and fleeting expression, manifests in almost all musical cultures as a mode of musical performance and creation. Prior to the 1970s, the field of musicology tended to regard improvisation as a “craft” rather than an “art” of composition. Case studies of improvisation began in the 1960s within ethnomusicology, primarily focusing on three categories: jazz, Indian art music, and Iranian music (Nettl, 2001). The focus on these musical styles also influenced the research methodologies for improvisation in other cultures. The methods of jazz improvisation are broadly divided into three categories: paraphrase improvisation, formulaic improvisation, and motivic improvisation. In practice, performers may use some or all of these types within a single improvisational performance, frequently overlaying them. Paraphrase improvisation is an ornamental variation on a theme or certain parts of a theme; formulaic improvisation constructs new material out of miscellaneous ready-made ideas; motivic improvisation builds new material through the development of a single ready-made idea (Kernfeld, 2001). The latter two types can respond to the theme or unfold independently of it. In addition to the aforementioned methods of improvisation, there are many idiomatic approaches during the improvisational process. Among them, modal improvisation is widely applied, which refers to improvising using ecclesiastical modes or to a non-diatonic scale from traditional or non-Western music, aiming to explore the melodic and harmonic possibilities of a set of pitches. Different improvisational methods are heavily intertwined, making it difficult to categorize them completely independently. Before the late 1950s, motivic improvisation appeared in jazz with far less frequency than paraphrase or formulaic improvisation. This was because jazz improvisation was previously expected to conform to an underlying theme; the given theme typically contained a functional progression unfolding at a harmonic rhythm of one, two, or four chords per measure, and the improvisation itself usually developed rapidly. Under these conditions, it was extremely difficult to systematically construct a motif without error.

Beginning in the late 1950s, new styles provided a more suitable framework within which motivic improvisation became more frequently used, rivaling paraphrase and formulaic improvisation in importance. Free jazz, modal jazz, jazz-rock, and other fusions of jazz and popular music began to phase out the characteristic themes of previous styles in favor of specific structures or simple bass lines and ostinatos. In any case, improvisers needed to follow fast-moving chord progressions while giving more attention to motivic improvisation. Furthermore, the repetition and development of motifs provide elements of coherence and stability, thereby reinforcing the theme. Genre is not a static, unmovable concept, but a reflection of the audience's assumptions and demands at a specific point in time. As the audience's assumptions about a musical genre change, the genre itself changes (Petrusich, 2021). Jazz improvisation usually unfolds within established harmonic frameworks and stylized vocabularies, as the overall genre and style of jazz shift across different periods, methods of improvisation exhibit pronounced differences.

Combining traditional music analysis with empirical data, this study draws upon the “Stream of Ideas”—an empirical-research-centered analytical framework established by scholars such as Martin Schütz for jazz improvisation, which can be applied to comparative stylistic studies (Schutz, 2012). Specifically, it quantifies improvisational behavior into data to serve as the foundation for analysis. This framework divides improvisation segments into a series of morphologically meaningful “basis ideas” : “Void” is used to name breaks between consecutive melodic phrases, unmated chords functioning as standardized accompaniment or sustaining tones at the end of an idea; “Lick” describes short melodic phrases with diversified rhythms based on Bebop-licks and fitted for specific chords; “Vague/Atmosphere” is assigned to musical phrases without recognizable context, direction, or form, used to create sound effects or ambience; “Shifting-phrase” describes repeated or periodic shifting between two tones or block chords; “Theme” is assigned to melodic phrases that pick up or vary the melody of the underlying tune; “Motif” is called melodic phrases or repeated figures that occur several times (repeated or varied) within an improvisation; “Line” describes a sequence of tones proceeding in small pitch intervals heading in a certain direction or shape; “Bellows” describes a two-voice movement proceeding in parallel motion or with one pitch sustained while the other moves; “Scissors” describes a two-voice movement extending in contrary motion. This method provides an objective way to compare the possibilities of different improvisations, displaying these known hypotheses in a quantitative manner rather than focusing solely on a single individual's performance. By integrating this research methodology, this paper will investigate the variations among the different jazz improvisation segments involved, visually demonstrating the specific differences in idea distribution and creative style across Monk, Shimon, and Suno, thereby providing tangible data and analytical references for GAI music analysis.

## 5 A comparative analysis of jazz improvisation excerpts

This study is an exploratory multiple-case study that compares three improvisational cases based on *Blue Monk*, a classic composition by jazz master Thelonious Monk. Unlike most of Monk's intricate compositions, this 12-bar blues track is accessible enough for even beginners to perform, making it a standard choice for jazz improvisation. Monk envisioned each new variation as a cohesive whole, yet equipped each with its own motivic development and distinct identity (Gioia, 2012). Starting from a jazz standard allows for a clearer observation of the entire jazz improvisational process. Such micro-improvisational acts, anchored in a classic text yet dynamically diverging and expanding on stage—effectively reconstruct the boundaries of the musical work in real time. This dynamic echoes a philosophical and musicological argument proposed by Brian Kane, who focuses on repetition and naming to formulate a realist view of the musical work in performance—an emergent, non-essentialist, and network-based ontology of the jazz standard, wherein “work-determinacy” is deemed sufficient rather than necessary (Kane, 2017). This ontology can be extended to the interactive processes between jazz and GAI. The essence of jazz is not bound to written notation, nor is the standard an immutable and fixed frame, different performers and media continuously shape the perceptual process. When participating in improvisation, GAI equals other agents in constructing this network of standards, thereby rewriting the very identity of the musical work.

In terms of sample selection, the three improvisational cases comprise Monk's own improvisational recording of *Blue Monk*, a live collaborative performance of this piece between the marimba robot Shimon and human musicians, and a musical piece generated by Suno based on the main melody of the track with jazz prompt instructions. Rather than deriving generalized statistical laws, these cases were chosen because, under the unified 12-bar blues framework, they precisely instantiate three quintessential technological paradigms across human cognition, physical robotics, and pure digital foundation models: Monk represents the pinnacle of human embodied cognition and jazz syntax, serving as the highest artistic benchmark for evaluating AI. Shimon, developed by the Center for Music Technology at the Georgia Institute of Technology, is a marimba-playing robot equipped with improvisational capabilities. Shimon utilizes Hidden Markov Models (HMM)—statistical techniques typically used in natural language processing—which have previously been applied to modal harmonic composition. In Shimon's model, the corpus is parsed into sequential chords, each reduced to its harmonic function with pitch representing scale degrees, and applied across varying combinations of pitch and duration. The resulting style-driven model governs the robot's real-time performance, analyzing incoming harmonic and melodic content and matching generated patterns against HMM sequences. Furthermore, Shimon applies Carol Krumhansl's pitch contour theory to match observed note frequencies with established major and minor tonal profiles (Miller, 2020). Thus, Shimon represents interactive AI driven by algorithms and physical hardware. Capable of real-time acoustic listening and physical mechanical limb expression, Shimon engages in real-time dialogue and physical interaction with human musicians on stage, exemplifying the application of Embodied AI in musical performance. Suno, developed by the American music technology company Suno Inc., converts text into original music using speech recognition, natural language processing, deep learning, and neural network technologies. Users can guide the AI to compose music via simple text prompts, achieving a personalized music production experience. Suno represents cutting-edge end-to-end audio foundation models based on big data Latent-space Learning. It bypasses written scores to generate complete, raw audio streams directly from text prompts, showcasing the capabilities of pure digital GAI in musical composition.

To enhance the reproducibility of this study, unified structural control criteria were established for the selection of all three improvisation segments, all analytical materials are based on the harmonic architecture of the same piece, thereby controlling for potential interference from background harmonic frameworks on how different improvising agents develop their ideas. Specifically, the detailed parameters and slice breakdown for the three experimental sample groups are as follows: (1) Monk: 3 consecutive full choruses (bars 13–49) of piano solo were selected from his classic recording segment; (2) Shimon: 3 non-consecutive full choruses played on marimba were selected (bars 16–28, 42–54, and 65–77); (3) Suno: 3 non-consecutive full choruses were selected from its generated audio, comprising two piano solo choruses and one trumpet solo chorus (bars 1–13, 25–37, and 71–83). The three sample groups maintain a high degree of isomorphism in both total duration and musical structure (each comprising three 12-bar blues structures), providing a standardized empirical foundation for the subsequent timeline-based sliced comparison of “Stream of Ideas” elements. Annotated via Timeliner software, the individual structural analyses for all three groups are detailed below.

Monk's work follows the structural division of traditional jazz (Figure 1), namely the classic pattern of Head—Solo—Head-out. On this basis, the expression of each instrumental part possesses its own ingenuity. In the piano solo section, Monk starts from a small, novel motif, continuously performing variations and developments, demonstrating immense creativity and expressiveness. In the drum solo section, it even breaks the previous conventional number of bars, and the mutual transitions highlight the profound tacit understanding and experience among the musicians. Monk's improvisation not only profoundly embodies the characteristics of motivic improvisation but also displays a high degree of freedom and individuality within the structural framework.

**Figure 1:**
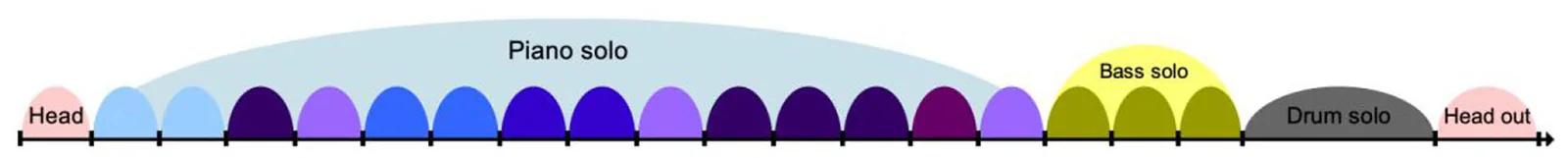
The structural layout of Thelonious Monk's improvisation excerpt.

Shimon's work with musicians similarly demonstrates interactive coordination among players; it performs alternating solos with the trumpet, saxophone, and trombone in turn, manifesting deep improvisational interaction through mutual dialogue (Figure 2). While improvising, Shimon behaviorally exhibits a “listening” gesture, responding to the other performers. Regarding its improvisational method, Shimon combines the characteristics of motivic and formulaic improvisation, mostly applying traditional harmonic progressions and fixed ostinato patterns. Compared to Monk's improvisation, Shimon slightly lacks variations in rhythm, dynamics, and tempo, its overall performance is more stable and conservative, lacking a certain breakthrough quality, but consequently conveying a stronger sense of structure.

**Figure 2:**
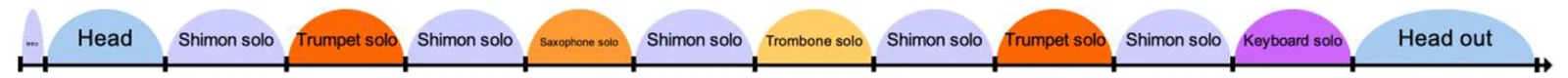
The structural layout of Shimon's improvisation excerpt.

Suno's work exhibits substantial differences compared to the former two, particularly in its improvisational techniques (Figure 3). Suno's improvisation predominantly utilizes paraphrase improvisation, resulting in a large amount of thematic repetition. Many of the improvisational techniques employed by Suno are not conventionally “jazzy,” such as the emergence of long bridge sections in the middle of the piece, as well as ascending and descending triad arpeggios resembling the cadenzas of classical works. Beyond this, it also employs certain elements of formulaic improvisation, occasionally relying on fixed pitch patterns and chord progressions within the melodic and harmonic structures, thereby displaying some traditional jazz improvisation characteristics. Compared to the former two, Suno's improvisation lacks a certain degree of coherence, its improvisation frequently imparts a sense of assemblage, as if multiple short, independent musical sections were pieced together, resulting in a performance that lacks fluidity and intrinsic unity. Notably, in the final head-out section, Suno ingeniously modulates to a higher register, breaking through the traditional framework and bringing an unexpected highlight to the performance.

**Figure 3:**
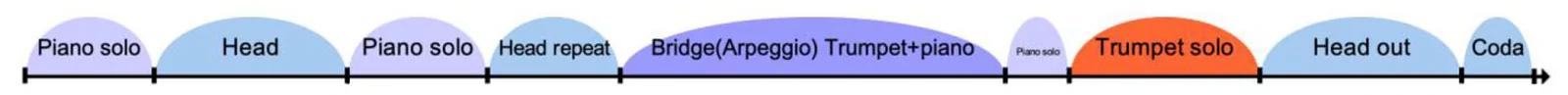
The structural layout of Suno's improvisation excerpt.

Following the structural analysis, specific improvisational excerpts were selected from the three cases to analyze the stylistic features and performance of various elements in different works using the “Stream of Ideas” analysis method. In the concrete analytical procedure, MIDI tracks were utilized to locate each “basis ideas” through their geometric clarity in pitch contours and vertical alignments (Figures 4–6). Simultaneously, qualitative calibrations were made against the original audio to reflect real auditory expressions in micro-timing and voice dynamics. While minor subjective cognitive variations regarding certain micro-categories may persist, the unified relative criteria guarantee the validity of cross-sectional comparisons. Given that the core objective of this study lies in highlighting the relative inter-subject differences rather than providing absolute musicological qualitative annotations, a cross-sectional comparison under unified relative standards remains effective in revealing the fundamental similarities and differences in structural generative logic among distinct improvising agents, thereby holding clear research significance and academic value.

**Figure 4:**
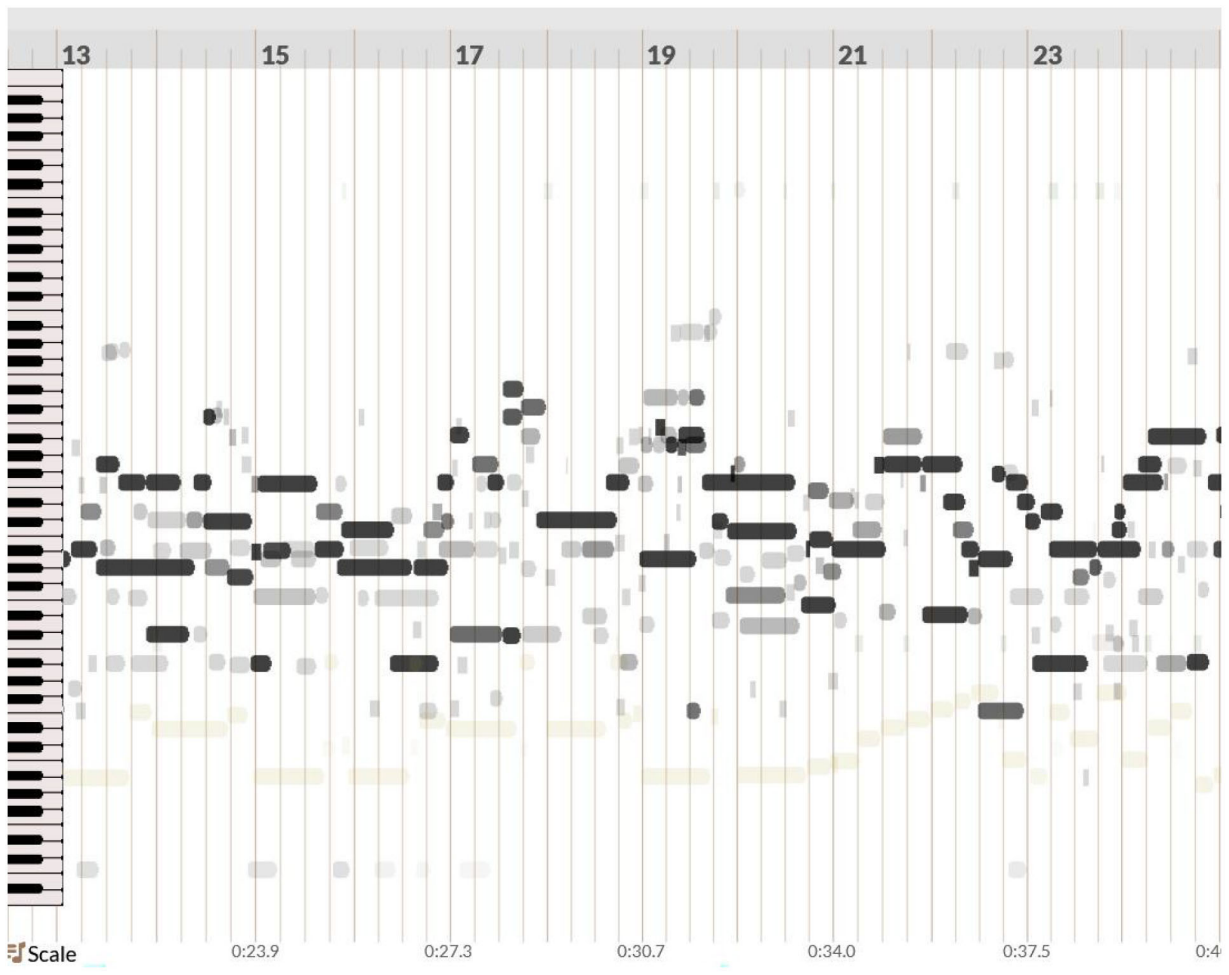
Sound distribution of Monk's improvisation excerpt.

**Figure 5:**
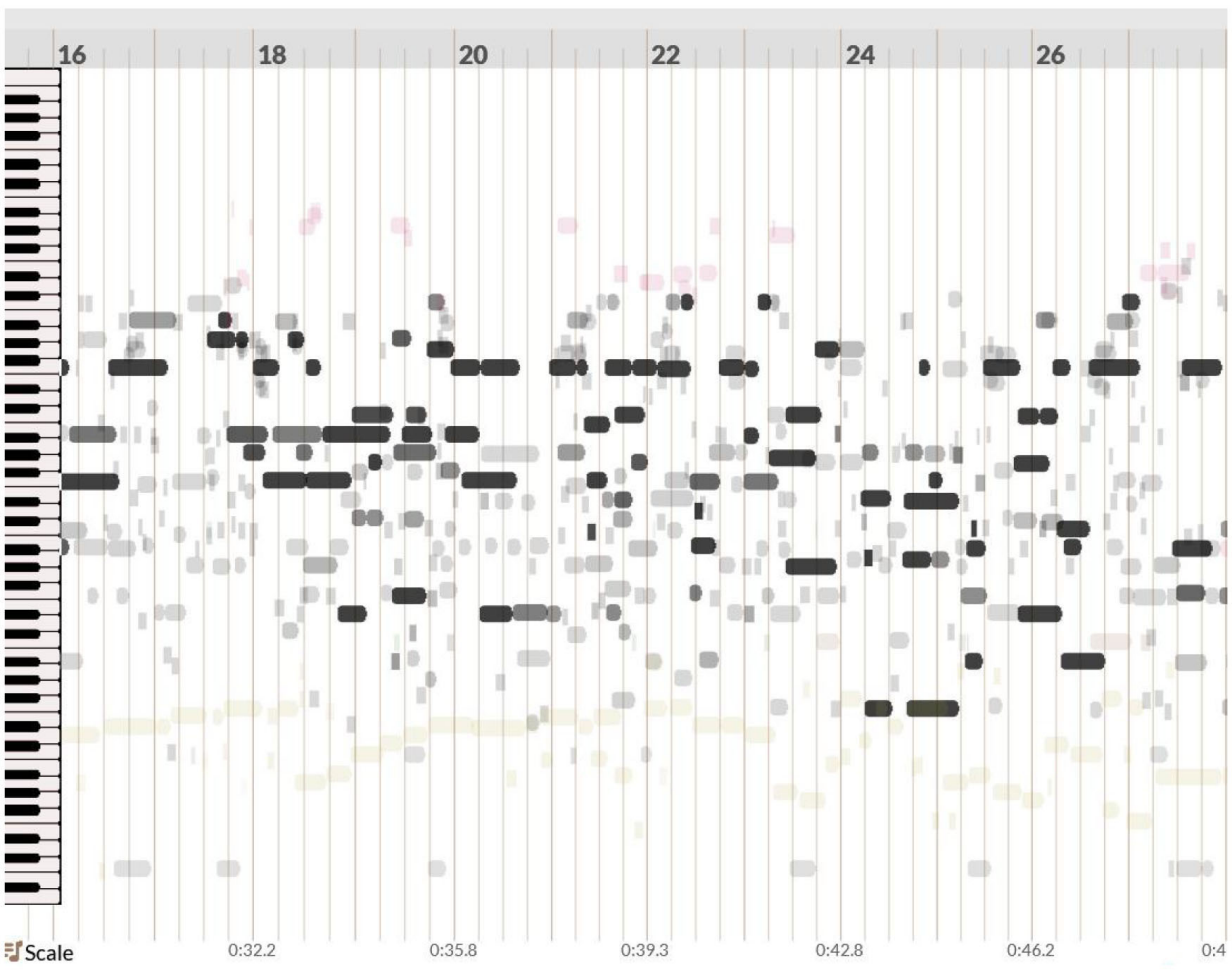
Sound distribution of Shimon's improvisation excerpt.

**Figure 6:**
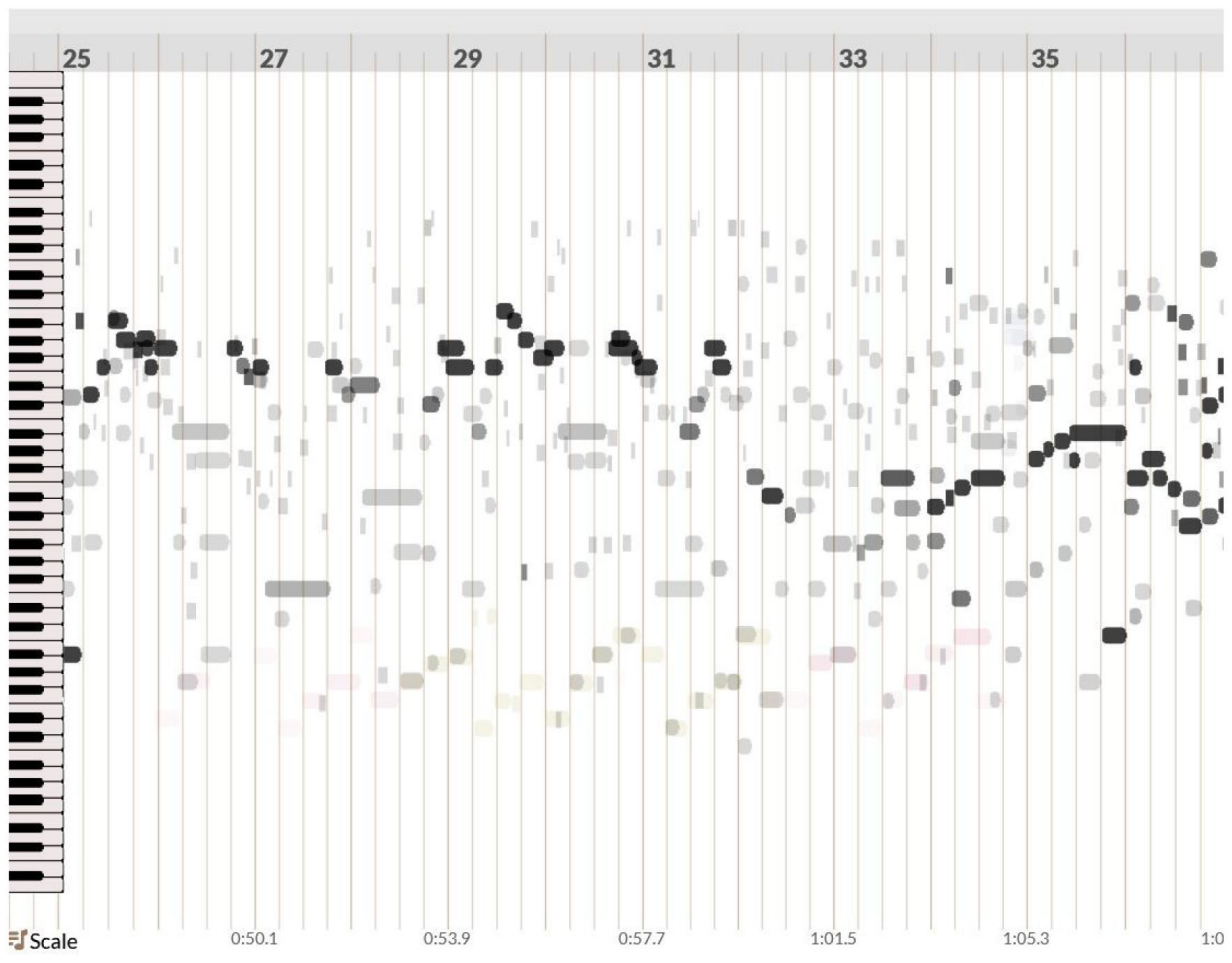
Sound distribution of Suno's improvisation excerpt.

Through a measure-by-measure statistical analysis of element frequencies across the cases (Figure 7), it can be intuitively observed that both Shimon and Suno demonstrate appropriate applications of core jazz stylistic features across various elements, including the organization5 of linear melodies, the use of classic licks, and the sense of phrasing breath and space (void). Combined with the aforementioned structural analysis, it can be concluded that both systems exhibit explicit jazz generation capabilities and possess a degree of technological agency in this domain. However, closer observation reveals that GAI still exhibits marked differences from Monk in the micro-level handling and expressiveness of these elements. In terms of applying “void”, Monk demonstrates an exceptionally strong sense of breath, frequently inserting rests throughout his solo passages to articulate phrases with vitality and grace, by contrast, Shimon and Suno display a relative scarcity of space, exhibiting a clear tendency toward note overload and sonic stacking. Regarding thematic and motivic development, the three entities favor distinct improvisational types and logics, Monk exemplifies “Motivic Improvisation,” using motifs to construct new materials while establishing mutual call-and-response relationships. Shimon embodies “Formulaic Improvisation,” virtually departing from the theme to compose nearly entirely new material within a modal framework. Suno leans toward “Paraphrase/Formulaic Improvisation,” with its improvisational passages featuring substantial borrowing and direct copying of the theme. In terms of arrangement and orchestration across the three cases, GAI's expressiveness in harmonic color and ensemble instrumentation is similarly inferior to Monk's live performance. Monk incorporates extensive variations such as substitute chords and modal interchange in his playing, whereas the harmonic colors of Shimon and Suno remain overly conservative and monolithic. Regarding overall ensemble arrangement, Monk's performance features significantly more dialogue and interaction among different instruments, accompanied by pronounced dynamic and textural shifts. Shimon and Suno show somewhat conservative performances, with noticeable deficiencies in transition and collaboration between instruments. Furthermore, their improvisational melodies mostly consist of newly generated thematic lines following prompt styles within fixed modal frameworks, exhibiting little deep-level call-and-response to the original theme. Although GAI can produce correct responses through algorithms and data models, its creation remains constrained by design and training datasets, lacking genuine creative dialogue. In contrast, Monk's performance is far more flexible and vivid, not only employing diverse harmonic and modal variations, but also creating richer musical layers through dynamic interactions among different instruments.

**Figure 7:**
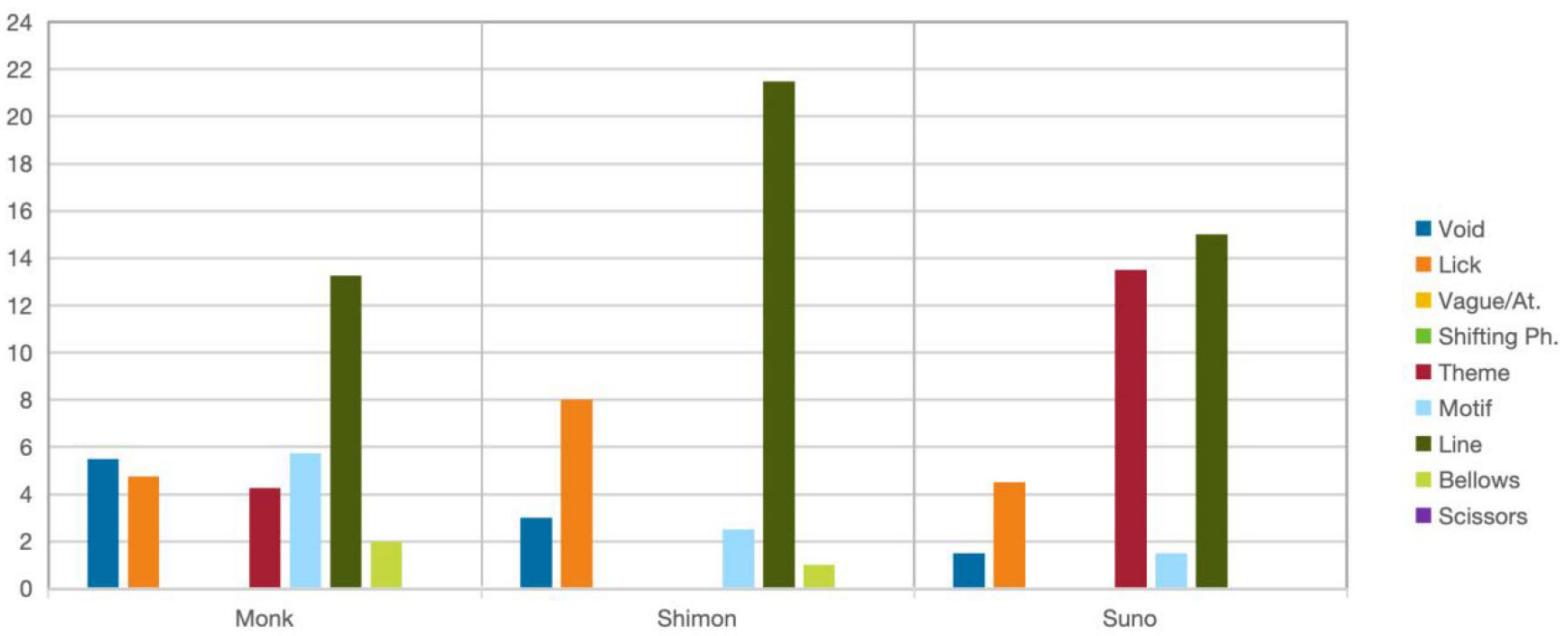
Frequency distribution of used “basis ideas” across the three cases.

## 6 The manifestation of GAI's agency as an actor in jazz improvisation

For jazz, repeating a form and then reversing it through a process of variation constitutes its core essence (Gates, 1988). Jazz improvisation inherently embodies several complex dialectical processes: spontaneity vs. established frameworks, modes of statement vs. existing vocabularies, and negotiation between the individual and the collective. It is precisely in reconciling the tension between innovation and tradition, and between spontaneity and organization, that performers strive to achieve the fusion of the individual and the collective, which resides at the core of jazz aesthetics (Louth, 2012). In improvisational music such as jazz, the interaction between the group and the individual profoundly shapes the ultimate composition and development of the music (Monson, 1996). Unlike a traditional symphony orchestra, a jazz band allows each musician to express themselves freely according to their preference, maintaining a receptive sensitivity toward the self-expressive playing of other musicians during this process. The complex state of harmony they form does not derive from performing a shared score, but rather from the individual's free expression built upon the free expression of others; the more expressive each jazz musician is, the more other musicians draw inspiration from it, being stimulated to achieve more brilliant results (Eagleton, 2007). As a highly interactive artistic practice, jazz improvisation provides a decentralized field of emergence for GAI. In this scenario, GAI is not merely a tool for creating music within it, but also participates in this continuous, dynamic process. Whether engaging in real-time acoustic interaction with human musicians or integrating and processing present instructions and historical corpora, the agency of the technological actor directly cuts into the generation of concrete musical facts.

In the Western philosophical tradition, most philosophers maintain a subject-object dualism (Blackburn, 2016), strictly restricting agency as an exclusive privilege of humans. However, the autonomous capacity for action that emerges from GAI under interactive states poses a fundamental challenge to this traditional, instrumentalist “human-machine dichotomy”. It transcends traditional AI's instrumental dependence on mechanical programming functions, displaying a distributed, non-linear manifestation of agency within the dynamic network of jazz improvisation. GAI and its performance activities with human musicians can be viewed as an assemblage of actors. To borrow Andrew Pickering's expression, this constitutes a “dance of agency” (Pickering, 2010). Within the actor-network, GAI acts as a non-human actor capable of mimicking human composition; its agency is primarily reflected through the design of programming and datasets, depending upon frameworks and goals engineered by humans, and it remains constrained by them. In Shimon's improvisation, the improvisational responses it makes depend on the musician's immediate expression in the preceding moments, the network of human-machine collaboration is in a state of constant dynamic construction. Suno relies even more heavily on instructions input by human actors, the more detailed and guiding the prompt, the more the resulting work aligns with the expected style. It is precisely because of this dynamic characteristic that both listening and analysis are fraught with uncertainty. This mode of collaboration mirrors the improvisational tradition of jazz, where this agency does not manifest as a single action but as a sustained driving force running through the entire creative process.

To avoid conceptual over-generalization, however, the analytical boundaries of GAI's agency must be strictly specified within the framework of ANT. Specifically, this study distinguishes between metaphorical agency and actual or technical agency. At the level of aesthetic perception and auditory discourse, AI manifests a form of “metaphorical agency”, human musicians or listeners often attribute an intentional improvisational role to the AI based on its sonic outputs. At the computational and algorithmic level, however, the AI system possesses merely an “actual or technical agency.” That is, it lacks human-like intentionality, subjective experience, and autonomous human-style creativity, its improvisational decisions are fundamentally weight calculations and parameter responses within high-dimensional probability spaces generated by deep learning models. Clarifying this conceptual boundary enables us, when analyzing human-machine improvisation, to both acknowledge the improvisational metaphors that AI brings to human listeners in sonic forms and maintain a clear-eyed critique of its algorithmic nature and mathematical logic, thereby avoiding the academic pitfall of technological anthropomorphism. From the perspective of ANT, Shimon and Suno can be viewed as non-human actors whose agency is embodied in how they reshape the process of musical practice, alter the pathways of signal translation, and exert action constraints on human-machine interactive systems (Translation and Mediation), rather than deriving from any internal, psychological sense of “intentionality” or “machine imagination.”

## 7 The material construction and critical reflection of GAI in the sociology of music

Within the ideological horizon of ANT, the agency of GAI manifests an interactive and dependent dual characteristic. Although GAI algorithms possess the capacity to generate music and can create creative improvisations based on inputted parameters, their generative process is invariably constrained by computational power, data models, programming frameworks, and material mediums—a limitation that essentially highlights its lack of embodiment and inherent restrictions. Musical perception and cognition are embodied and situated activities; this implies that they crucially depend upon the physical constraints and enablements of our sensorimotor system, as well as the ecological and socio-cultural environments that shape our capacities for music listening and creation (Iyer, 1998). For instance, in this case, Shimon's composition was primarily anchored in HMM, with technological iterations, this model exhibits generative limitations when confronting complex jazz improvisation, as it can only capture short-term sequential dependencies. Furthermore, the live performance technique of the marimba robot Shimon is restricted by the physical movement limits of its mechanical fingers, which distinguishes the agency of GAI from the autonomous freedom of human creators. The agency of GAI resembles a collaborative consequence among multiple actors; this restrictive nature renders its role in jazz improvisation more akin to an instrumental assistance rather than completely autonomous and free creation. Beyond this, the transmission of music and the sensations it evokes depend not merely on compositional techniques but are also embodied in the mediums and modes of expression. In terms of timbre and sonic quality, the reason synthesized music is perceived as less engaging than human live performance is that real acoustic instruments offer “expressive resources” that provide far richer variation and stimulation to the auditory cortex (López de Mántaras and Arcos, 2002). This cognitive stratification conditioned by material mediums demonstrates that if technological artifacts are to truly transcend the boundaries of mere instrumental assistance, their primary bottleneck lies no longer in the physical output of generative algorithms, but in whether they possess the subjective aesthetic capacity to deconstruct these complex expressive resources.

If GAI is to truly compose music, it must first be taught aesthetics, which poses an ultimate challenge. Musical aesthetics is a complex, hybridized process wherein emotions, mediums, and other variables directly or indirectly guide the purpose and outcome of creation. There is no independent subject, but only a collective assemblage of enunciation (Deleuze and Guattari, 1987). Driven by aesthetic intentionality, “errors” occasionally transform into unexpected surprises. Jazz improvisation, at its core, is a delicate equilibrium that simultaneously harbors the uncertainty of disorder and loss of control, while encapsulating the transformative power to breach boundaries. Consequently, improvisation provides an experimental space where performers must responsively navigate various contingencies and swiftly recalibrate their performance direction, translating “errors” into refreshing creative expressions. This spirit of adventure within improvisational creation constitutes the very fountainhead of jazz's vigorous vitality. This improvisational adventure within states of disorder inherently redefines the algorithmic alignment between human and non-human. To endow generative architectures with the aesthetic potential required to navigate such experimental spaces, technological artifacts must be anchored in deeper ethical and metaphysical coordinates. As Elon Musk conceptualized in a recent dialogue on computing and the future of intelligence, truth, curiosity, and beauty should serve as the core dimensions guiding the development of non-human intelligence (Musk, as cited in Diamandis, 2026). Only through the combined interplay of rule constraints and aesthetic guidance can GAI transcend mere collage and replication, becoming an effective tool for collaborative creation with humans.

Through this project's comparative study of human musicians and GAI in jazz improvisation, it becomes clear that despite its rapid evolution and iteration, GAI at the current stage remains far from entirely convincing, human listeners can still distinctively identify works produced in a characteristic GAI style. This echoes Latour's concept of the black box, no matter how controversial their history, how complex their inner workings, how large the commercial or academic networks that hold them in place, only their input and output count (Latour, 1987). Perhaps when the output of GAI is acknowledged by musicians and audiences as “good jazz,” this black box succeeds, people no longer care how it was computed, but only whether its performance is pleasing to the ear. The optimal state of our relationship with GAI might resemble Heidegger's state of readiness-to-hand, wherein it possesses a mode of being-in-itself rather than merely sitting there as presence-at-hand, rendering it ready for manipulation and utilization in the broadest sense. Our ordinary dealings do not tarry primarily with the equipment itself but with the work—the thing to be produced at the time, the work itself is also something ready-to-hand. The work bears with it a whole referential totality of involvements, within which the equipment is encountered (Heidegger, 1962).

The agency demonstrated by GAI in the generation of jazz improvisation reveals a complex sociotechnical interactive network model; musical computer programs, like any other text, are neither objective nor universal, but represent the specific thoughts of their creators. As ideas about the nature and function of music become embedded in software-based musical systems and compositional structures, interactions with these systems often reveal the characteristics of the “community of thought” and culture that produced them (Lewis, 2000). This not only encompasses the creative potential of the technology itself, but also profoundly reflects the penetration and control exerted by capital market forces over the creative process—elements that are difficult for ANT to balance. In his critique of ontological and materialist approaches to sound, Campbell deploys ANT as an alternative to these methods, while simultaneously pointing out certain limitations of ANT in handling critique and abstract themes; for instance, the ANT approach may struggle to account for the political and ideological forms that produce structures of exclusion (Campbell, 2021). Through the performance of GAI in jazz improvisation, it is evident that as a technical actor, its generative process is co-conditioned by algorithms, datasets, and commercial demands, thereby invisibly shackling it within established commercial structures and technological frameworks. Born also notes that introducing ANT to music or pursuing theories of musical mediation must account for the following deficiencies, the necessity for a more nuanced empiricism in ethnographic and historical studies of music, the demand to cross scales in analysis with reference to the social, temporal, and material mediations of music while linking them to questions of power, the challenge of theorizing subjectivity alongside aesthetic and emotional processes and the question of ontology (Born and Barry, 2018).

As Born warns, expanding the focus from purely technical networks to questions of power and subjectivity reveals that GAI's intervention in jazz improvisation gives rise to far more than a methodological dispute over agency, it poses a host of urgent socio-ethical propositions. At the levels of data transparency and authorship, the absence of psychological intentionality and consciousness in AI creates a logical dilemma: its outputs can neither be straightforwardly attributed to autonomous machine creation nor accommodated within traditional frameworks of author-centrism. Taking Suno as an example, despite utilizing vast amounts of musical data for training, the system operates as a data black-box, leaving users with little control or understanding regarding dataset selection, processing methodologies, or parameter tuning, thereby restricting its application in personalized, deep artistic expression. Beyond individual agency, artistic creation remains deeply embedded in historical and community-based social networks. When commercial algorithms commercialize shared jazz vocabularies as proprietary technical assets, recognizing the collective cultural contributions of historical musicians in training datasets becomes an urgent ethical imperative, calling for a dynamic balance between protecting innovation and honoring cultural lineage rather than simple disputes over ownership. This algorithmic mediation extends directly into modes of production and labor structures, where intensified technical control threatens to homogenize creative practices. As generated works increasingly conform to market standards and commercial expectations, the distinct personality and emotional investment of artists risk being diluted, curtailing stylistic diversity. While AI's data-extraction capabilities allow it to efficiently execute standardized, assembly-line tasks such as background arranging, undeniably squeezing the commercial survival space for foundational musical labor. This very commodification conversely underscores the irreplacability of live human performance, where embodied perception forms a core value beyond algorithmic computation. Concurrently, on the terrain of cultural appropriation and political ideology, after jazz music, epitomized by figures like Monk, carries profound memories of racial history and cultural resistance peculiar to African-descended communities. Commercial AI algorithms frequently reduce these living cultural symbols to abstract probability models, stripping them of their original socio-historical contexts and enacting a form of covert cultural appropriation. Ultimately, interrogating these ethical dilemmas serves as a critical reminder that technological advances must prioritize human creative freedom, labor dignity, and cultural vitality. The definitive purpose of technology lies not in substituting algorithmic calculation for the human aura, but in re-establishing humanity as the primary artistic subject and cultural bearer within an emerging human-machine symbiosis.

## 8 Conclusion

The penetration of GAI has quietly altered human life, and a frequently discussed question of subjective authenticity is: what exactly counts as human innovation? With the rapid development of GAI, the boundaries between machine and human grow increasingly blurred. GAI can generate artworks through complex algorithms and models, while these pieces seemingly possess creativity, they are fundamentally anchored in pattern recognition and representation based on massive datasets, rather than human-like self-expression or a unique interpretation of the world. These output behaviors are frequently questioned by people: does this constitute genuine creation? This remains an open question, yet it is undeniable that if a work can cause an individual to vibrate with emotion, who created it seems to become far less important.

The contemporary musical landscape is being dramatically reshaped, and we frequently feel doubt or unease regarding where we stand and the transformations we confront. In this process, the actor-network provides a unique mode of understanding and a pathway for intervention. Following the actors, clear of preconceptions and pre-established frameworks, allows for a deeper understanding of the entire network, actors are not merely human individuals, but any entity that exerts an effect within the network. GAI, which possesses a high degree of interactivity with humans, is no longer treated merely as a tool or a passive technology, but as an actor endowed with agency. Jazz improvisation is a cultural practice rich in innovation, serving as an ideal vehicle for a profound understanding of how technology exerts its effects upon the creative process. Through the actor-network, the agency of actors is reconstructed, and control under the power of capital markets looms large across various facets. This alerts us and makes it meaningful to begin rethinking the essence of creation alongside pluralism within jazz improvisation. GAI's agentic participation in jazz improvisation constitutes not only an output but, more importantly, a feedback loop.

As a non-human actor, GAI, along with multiple actors such as musicians and listeners, jointly constitutes a dynamic and complex network, co-creating through mutual interactions, integrating the past while expressing the present. The more we find ourselves in the midst of such violent upheavals of technological paradigms, the more we need to prudently question the ontological positioning of humans in the future artistic ecology. Facing this transformative prospect, the core imperative for human creators is no longer to cling to the absolute myth of author-centrism, but to transcend the limitations of instrumentalism, enduringly sustaining a reflective, authentic perception and critical introspection. As traditional boundaries are shattered and actor-networks are reorganized, the relationship between technology and art, and between machine and human, becomes an increasingly prominent proposition of our time. Wherever our gaze lands, we find new interpretations within the academic horizon, representing an inevitable momentum toward the mutually constitutive symbiosis of human art and intelligent technology, rooted in the present and facing the future.

## Data Availability

The original contributions presented in the study are included in the article/supplementary material, further inquiries can be directed to the corresponding author.
